# Contribution of S6K1/MAPK Signaling Pathways in the Response to Oxidative Stress: Activation of RSK and MSK by Hydrogen Peroxide

**DOI:** 10.1371/journal.pone.0075523

**Published:** 2013-09-18

**Authors:** Anna Siebel, Monica Cubillos-Rojas, Roberto Christ Santos, Taiane Schneider, Carla Denise Bonan, Ramon Bartrons, Francesc Ventura, Jarbas Rodrigues de Oliveira, Jose Luis Rosa

**Affiliations:** 1 Departament de Ciències Fisiològiques II, Campus de Bellvitge, Institut d’Investigació Biomèdica de Bellvitge (IDIBELL), Universitat de Barcelona, L’Hospitalet de Llobregat, Barcelona, Spain; 2 Laboratório de Neuroquímica e Psicofarmacologia, Faculdade de Biociências, Pontifícia Universidade Católica do Rio Grande do Sul, PUCRS, Porto Alegre, Rio Grande do Sul, Brazil; 3 Laboratório de Biofísica Celular e Inflamação, Faculdade de Biociências, Pontifícia Universidade Católica do Rio Grande do Sul, PUCRS, Porto Alegre, Rio Grande do Sul, Brazil; 4 Laboratório de Microbiologia Clínica, Ciências da Saúde, Centro Universitário Franciscano, UNIFRA, Santa María, Rio Grande do Sul, Brazil; University of Southern California Keck School of Medicine, United States of America

## Abstract

Cells respond to different kind of stress through the coordinated activation of signaling pathways such as MAPK or p53. To find which molecular mechanisms are involved, we need to understand their cell adaptation. The ribosomal protein, S6 kinase 1 (S6K1), is a common downstream target of signaling by hormonal or nutritional stress. Here, we investigated the initial contribution of S6K1/MAPK signaling pathways in the cell response to oxidative stress produced by hydrogen peroxide (H_2_O_2_). To analyze S6K1 activation, we used the commercial anti-phospho-Thr389-S6K1 antibody most frequently mentioned in the bibliography. We found that this antibody detected an 80-90 kDa protein that was rapidly phosphorylated in response to H_2_O_2_ in several human cells. Unexpectedly, this phosphorylation was insensitive to both mTOR and PI3K inhibitors, and knock-down experiments showed that this protein was not S6K1. RSK and MSK proteins were candidate targets of this phosphorylation. We demonstrated that H_2_O_2_ stimulated phosphorylation of RSK and MSK kinases at residues that are homologous to Thr389 in S6K1. This phosphorylation required the activity of either p38 or ERK MAP kinases. Kinase assays showed activation of RSK and MSK by H_2_O_2_. Experiments with mouse embryonic fibroblasts from p38 animals’ knockout confirmed these observations. Altogether, these findings show that the S6K1 signaling pathway is not activated under these conditions, clarify previous observations probably misinterpreted by non-specific detection of proteins RSK and MSK by the anti-phospho-Thr389-S6K1 antibody, and demonstrate the specific activation of MAPK signaling pathways through ERK/p38/RSK/MSK by H_2_O_2_.

## Introduction

Reactive oxygen species (ROS) function as important physiological regulators of intracellular signaling pathways [1]. High ROS levels are associated with diseases such as neurodegeneration, atherosclerosis, chronic inflammation, diabetes or cancer [1-4]. An increase in ROS is also observed with age, probably caused by the accumulation over time of free radicals from aerobic metabolism and linked to a decreased antioxidant capacity and/or mitochondrial dysfunction [1,5]. The emerging role of ROS in physiological and pathophysiological processes demonstrates the importance of understanding the cell signaling pathways involved in redox signaling [1,3,6].

The mitogen-activated protein kinase (MAPK) signaling pathways allow cells to interpret a wide range of external signals and respond by generating a plethora of different biological effects. Members of the MAPK family, including extracellular signal-regulated kinases (ERK), c-Jun N-terminal kinases (JNK) and p38, are activated by ROS. The activation of these kinases usually regulates the expression of a variety of genes involved in survival, proliferation or cell death, depending on the stimulus and the cell-type studied [1,3,7].

The ribosomal protein S6 kinase 1 (S6K1) is a common downstream target of signaling by hormones and nutrients. S6K1 is a substrate of the mammalian target of rapamycin (mTOR) complex 1 (mTORC1). This complex is a Ser/Thr kinase that regulates S6K1 activation through its phosphorylation at Thr389 (T389). Activated S6K1 regulates the phosphorylation of other substrates such as the ribosomal protein S6 to promote protein synthesis, cell growth and cell proliferation [8-10]. In recent years, several studies have also involved S6K1 in the response to oxidative stress. Thus, whereas some authors propose that mTOR inhibition is required for H_2_O_2_-induced cell death [11], others demonstrate that the mTOR/S6K1 pathway is not responsible for this effect [12]. In some cases, S6K1 phosphorylation was observed [12,13], whereas in others a decrease in this phosphorylation was reported [11,14,15]. These apparently controversial findings have been justified by the complexity of the pathways involved and by the function of these pathways possibly depending on the cell type, H_2_O_2_ dose and duration of the stress signal [12].

S6K1 activation is measured by the increase of its phosphorylation at T389 and/or by the phosphorylation increase of its substrate, the ribosomal protein S6, at S235/S236. Thus, antibodies against these phosphorylated residues are a valuable tool for analyzing S6K1 activation. The specificity of these antibodies is crucial to interpretation of the data. S6K1 is member of a family of serine /threonine kinases named AGC. Other members of this family, such as the mitogen- and stress-activated kinases (MSK) and the p90 ribosomal S6 kinases (RSK), show a high degree of homology, in particular a serine residue within the hydrophobic motif of the RSK and MSK proteins [16]. Previous studies have shown the cross-reaction of anti-phosphorylated-T389 (P-T389)-S6K1 antibody with phosphorylated RSK and MSK proteins and that activation of these kinases also regulate the phosphorylation of the ribosomal protein S6 at S235/S236 [17].

In response to oxidative stress, MAPK signaling pathways are activated; contradictory data have been reported for the S6K1 signaling pathway. We asked whether under these conditions the anti-P-T389-S6K1 antibody detected RSK and MSK proteins and could be a motif to misinterpret these findings. In the present study, we showed that S6K1 is not involved in the fast response to incubation with H_2_O_2_ and that the anti-P-T389-S6K1 antibody detected the phosphorylation of RSK and MSK proteins by H_2_O_2_ in a p38- and ERK-dependent manner.

## Materials and Methods

### Reagents

Insulin, wortmannin, rapamycin and anti-P-ERK1/2 antibody (Sigma-Aldrich); hydrogen peroxide solution (H_2_O_2_) (Panreac); U0126 and SB203580 (Calbiochem); anti-mTOR, anti-P-T389-S6K1 (1A5), anti-P-S380-RSK, anti-P-S376-MSK, anti-P-S235/236-S6, anti-S6 (54D2) and anti-PT180/Y182-p38 antibodies (Cell Signaling Technology); anti-MSK, anti-S6K1 (C-18) and anti-RSK1 (C-21) antibodies (Santa Cruz Biotechnology, Inc.); Alexa Fluor 488, Alexa Fluor 546, TO-PRO3 (Molecular Probes); anti-P- H2AX antibody, Immobilon-P PVDF transfer membrane (Millipore Corporation); siRNA used: mTOR (CCCUGCCUUUGUCAUGCCUdTdT), S6K1 (GGGGGCUAUGGAAAGGUUUdTdT), RSK1 (**GCUAUACCGUCGUGA**-GAUCdTdT), RSK2 (GGAGGAGAUUAACCCACAAdTdT), MSK1 (**GGAACUGG**-AGCUUAUGGAAdTdT), MSK2 (UUGCACAUGAUCUCGGCCGdTdT) and non-targeting control (UAGCGACUAAACACAUCAAdTdT).

### Cell culture and transfections

WT and p38α-deficient MEFs were a gift from Dr. A. Nebreda (Institute for Research in Biomedicine, Barcelona, Spain) [18]. All cell lines were cultured at 37°C in Dulbecco’s Modified Eagle medium (DMEM) (Gibco), containing 10% fetal bovine serum. siRNA transfections were carried out in MCF7 cells with the calcium phosphate transfection system. For experiments with insulin, cells were deprived of serum overnight and then incubated with 200 nM insulin for 30 min. For experiments with H_2_O_2_, cells were treated with 0.4 mM H_2_O_2_ for 30 min, without overnight serum deprivation. The specific inhibitors were added 60 min before the treatment with H_2_O_2_ or insulin at a final concentration of 20 nM rapamycin, 100 nM wortmannin, 5 µM U0126 and 5 µM SB203580.

### Cell lysate and immunoblotting

Previously treated cells were lysed in CHAPS lysis buffer (10 mM Tris–HCl, pH 7.5, 100 mM NaCl, 0.3% CHAPS, 50 mM NaF, 1 mM sodium vanadate, 1 mM phenylmethylsulfonyl fluoride, 5 µg/ml leupeptin, 5 µg/ml aprotinin, 1 µg/ml pepstatin A, 50 mM β-glycerophosphate, 100 µg/ml benzamidine) for 1 h at 4°C and equal amounts of proteins were separated by electrophoresis. To analyze simultaneously large and small proteins in the same gel, we used Tris-Acetate PAGE systems [19]. After running the gel, the proteins were transferred to PVDF membranes and viewed by immunoblotting, as described elsewhere [17]. Band intensities were analyzed with a gel documentation system (LAS-3000 Fujifilm). Protein levels were standardized with respect to mTOR or Ran levels and expressed as a percentage of controls.

### Confocal microscopy

MCF-7 cells were fixed with 4% paraformaldehyde for 20 min at room temperature (RT). The cells were blocked and permeabilized with 10% fetal bovine serum and 0.1% Triton X-100 in PBS for 2h. The primary antibodies, anti-PT180/Y182-p38 (1:200), anti-P-S380-RSK (1:50), anti-P-ERK1/2 (1:200) and anti-P- H2AX (γH2AX) (1:500), were incubated at 4°C overnight; and the secondary antibodies, at RT for 2h. Nuclei were stained with TO-PRO-3 and the cells were examined by laser confocal microscopy.

### Immunoprecipitations and kinase assay

Lysates from MCF7 cells were immunoprecipitated with anti-RSK or anti-MSK antibodies. Lysis and immunoprecipitation were carried out in a buffer containing 40 mM Hepes, pH 7.5, 120 mM NaCl, 50 mM NaF, 0.3% CHAPS and the above protease inhibitors. Immunoprecipitates were washed three times with lysis buffer and once with kinase buffer (30 mM Tris-HCl, pH 7.5, 10 mM MgCl_2_, 1 mM DTT). The kinase assay was performed as previously described [17] in kinase buffer using GST-S6 as substrate (3 µg substrate per assay) in the presence or absence of ATP (500 µM), during 30 min at 30°C. Reactions were stopped in ice with sample buffer and analyzed by immunoblot with anti-P-S235/S236-S6 antibody to detect the incorporation of phosphate. Band intensities were analyzed with a gel documentation system (LAS-3000 Fujifilm).

### Statistical analysis

The results are expressed as mean ± SEM. Data were analyzed by one-way ANOVA followed by Dunn’s post-hoc test.

## Results

### Phosphorylation of p85 and S6 ribosomal proteins in response to H_2_O_2_

**Figure 1:**
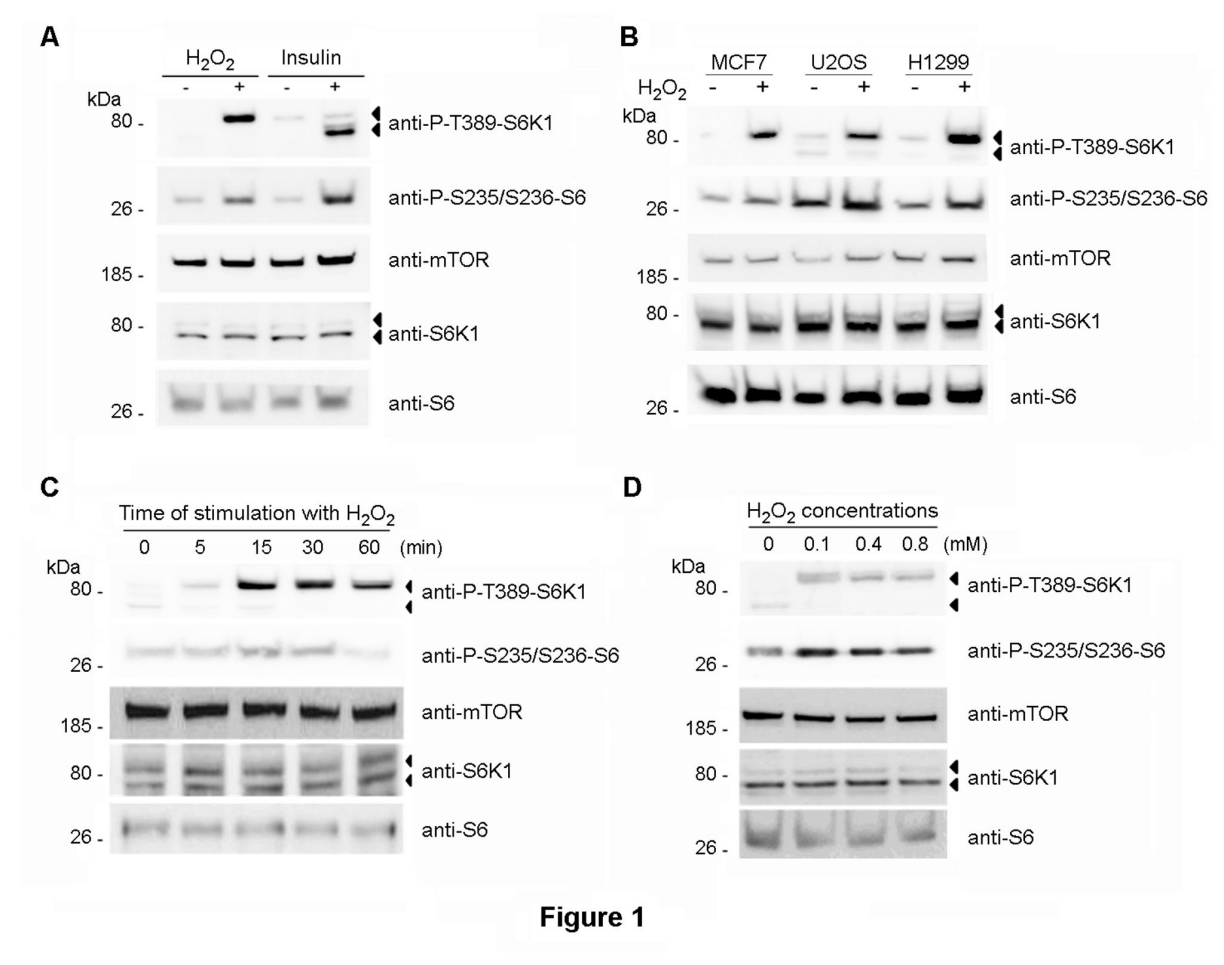
Phosphorylation of p85 and S6 ribosomal proteins in response to H_2_O_2._. Human cells were treated with 0.4 mM H_2_O_2_ for 30 min (A,B) or deprived of serum overnight and then stimulated with 200 nM insulin for 30 min (A). Experiments of time and dose course were performed in MCF7 cells with 0.4 mM H_2_O_2_ (C) or for 30 min (D), respectively. Cell lysates were analyzed by Western blot with the indicated antibodies. Molecular weight markers are indicated on the left.

To study S6K1 regulation in response to oxidative stress, we analyzed by Western blot the phosphorylation of endogenous S6K1 by H_2_O_2_, using a commercial monoclonal anti-P-T389-S6K1 antibody (1A5, Cell Signaling Technology). MCF7 cells were incubated with H_2_O_2_ for 30 min. We used these human cells because they have been extensively used to study the response to oxidative stress. As a positive control of S6K1 activation, MCF7 cells were stimulated with insulin after overnight serum deprivation. As shown in Figure 1A, phosphorylation of endogenous S6K1 p70 isoform was detected by Western blot with anti-P-T389-S6K1 antibody after 30 min of treatment with insulin. No variation in p85 isoform phosphorylation was detected in this cell line with insulin treatment. Unexpectedly, after treatment with H_2_O_2_, we detected an increase in a band with similar mobility to S6K1 p85 isoform that we named p85 protein. No increase was seen in p70 isoform phosphorylation. With both treatments, an increase in the phosphorylation of the ribosomal S6 protein was observed. These treatments did not modify endogenous levels of S6K1 or S6 proteins (Figure 1A). Levels of other proteins involved in the S6K1 signaling pathway such as mTOR were also unaltered (Figure 1A). These results were confirmed in other human cell types such as U2OS or H1299 (Figure 1B), indicating that the response to H_2_O_2_ is not restricted to one cell type.

Experiments of time and dose course confirmed the previous data. Thus, rapid (5 min) and specific phosphorylation of endogenous p85 protein was seen on incubation with various concentrations of H_2_O_2_ (Figure 1C/1D). Maximum effects were observed after 15-30 min incubation.

### The mTOR/S6K1 signaling pathway is not activated in response to the oxidative stress produced by H_2_O_2_

**Figure 2:**
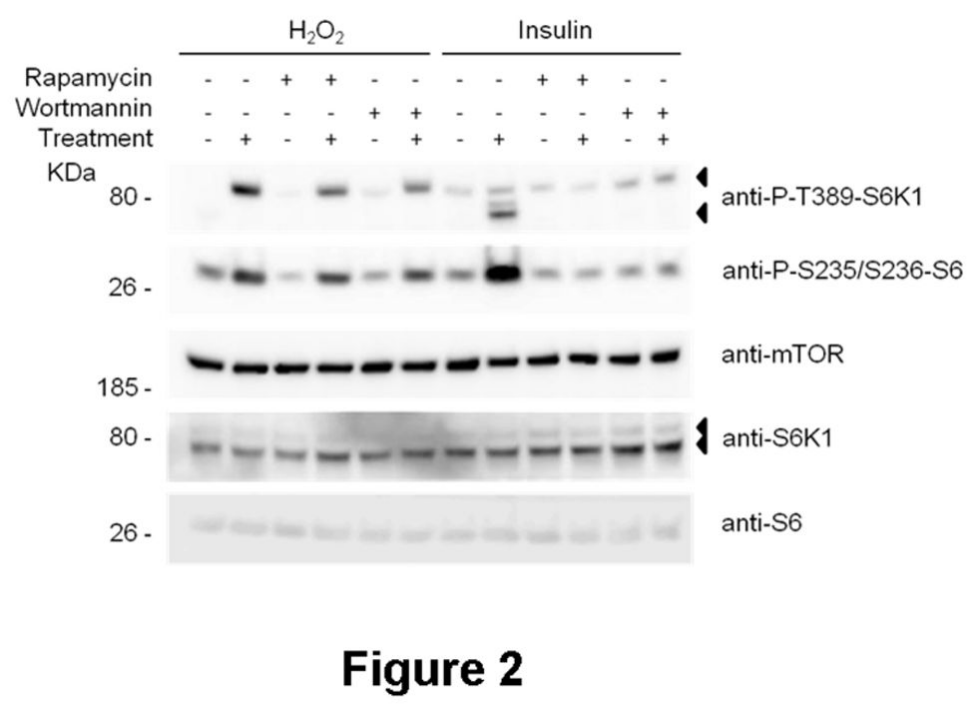
Effect of rapamycin and wortmannin on phosphorylation of p85 protein by H_2_O_2_. MCF7 cells were treated with 0.4 mM H_2_O_2_ for 30 min. Where indicated, MCF7 cells were pre-incubated with 100 nM wortmannin or 20 nM rapamycin for 60 min before treatment with H_2_O_2_. Cell lysates were analyzed by Western blot with the indicated antibodies. Molecular weight markers are indicated on the left.

To analyze the phosphorylation regulation of p85 protein, we performed experiments with H_2_O_2_ in the presence of known inhibitors of S6K1 activation (Figure 2). We observed that the phosphorylation of p85 protein was not significantly modified by rapamycin or wortmannin, inhibitors of mTORC1 and PI3K and mTOR kinases, respectively. S6 phosphorylation correlated with the increase in p85 phosphorylation and was not significantly modified by rapamycin and wortmannin. As control, in parallel experiments, cell stimulation with insulin confirmed the previously reported inhibition of S6K1 phosphorylation and S6 phosphorylation by rapamycin and wortmannin (Figure 2).

**Figure 3:**
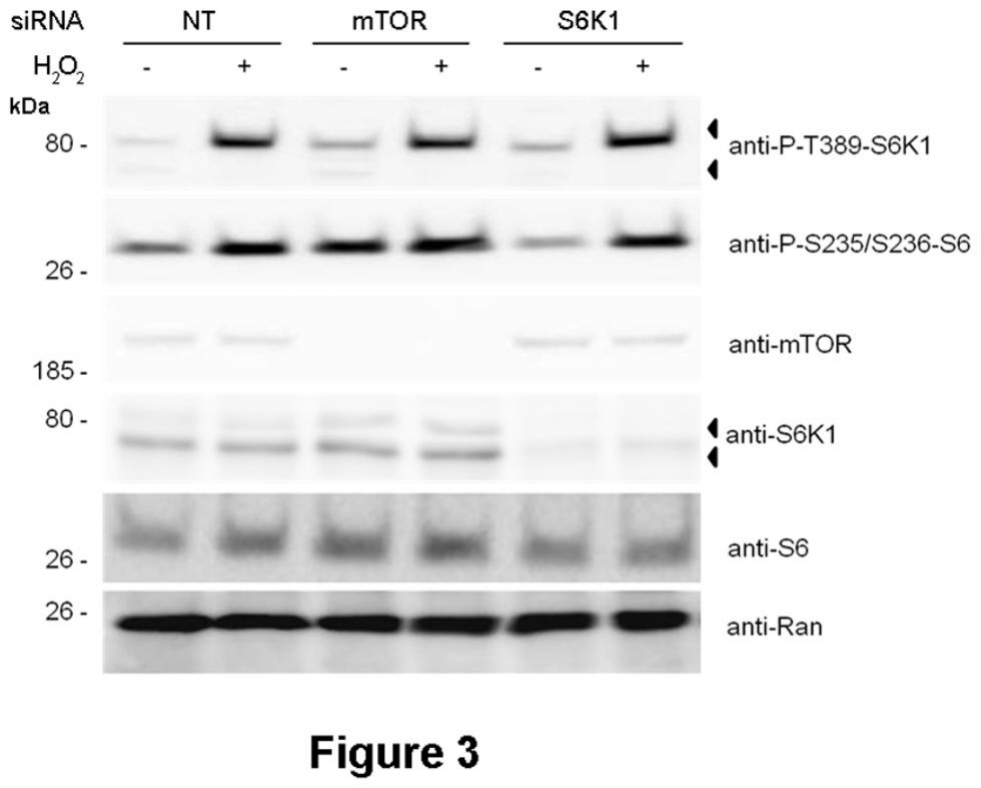
The mTOR/S6K1 signaling pathway is not activated in response to the oxidative stress produced by H_2_O_2_. MCF7 cells were transfected with the indicated siRNAs 72 h before treatment with 0.4 mM H_2_O_2_ for 30 min. Cell lysates were analyzed by Western blot with the indicated antibodies. Molecular weight markers are indicated on the left. NT means non-targeting control.

It was shown that rapamycin inhibits the phosphorylation of S6K1 isoforms by mTORC1 [10]. The above data seem to indicate that, in response to H_2_O_2_, this phosphorylation is not regulated by rapamycin (Figure 2) and suggest that another kinase might be involved in this regulation. To discard a role of mTOR protein, knockdown experiments were performed. As shown in Figure 3, in response to H_2_O_2_, the phosphorylation of endogenous p85 protein was not altered by the absence of mTOR, indicating that p85 protein is not a substrate of mTOR complexes.

We had previously reported that, in response to amino acids, anti-P-T389-S6K1 antibody recognized a phosphorylated p85 protein that was not S6K1 [17]. The similarity of these results with what we obtained with H_2_O_2_ led us to analyze whether the phosphorylated p85 protein was S6K1. To this end, knockdown experiments of S6K1 were performed. As shown in Figure 3, the phosphorylation of endogenous p85 protein was not altered by the absence of S6K1, indicating that the phosphorylated protein detected by the anti-P-T389-S6K1 antibody is not S6K1.

### Activation of the MAPK signaling pathways in response to the oxidative stress produced by H_2_O_2_

**Figure 4:**
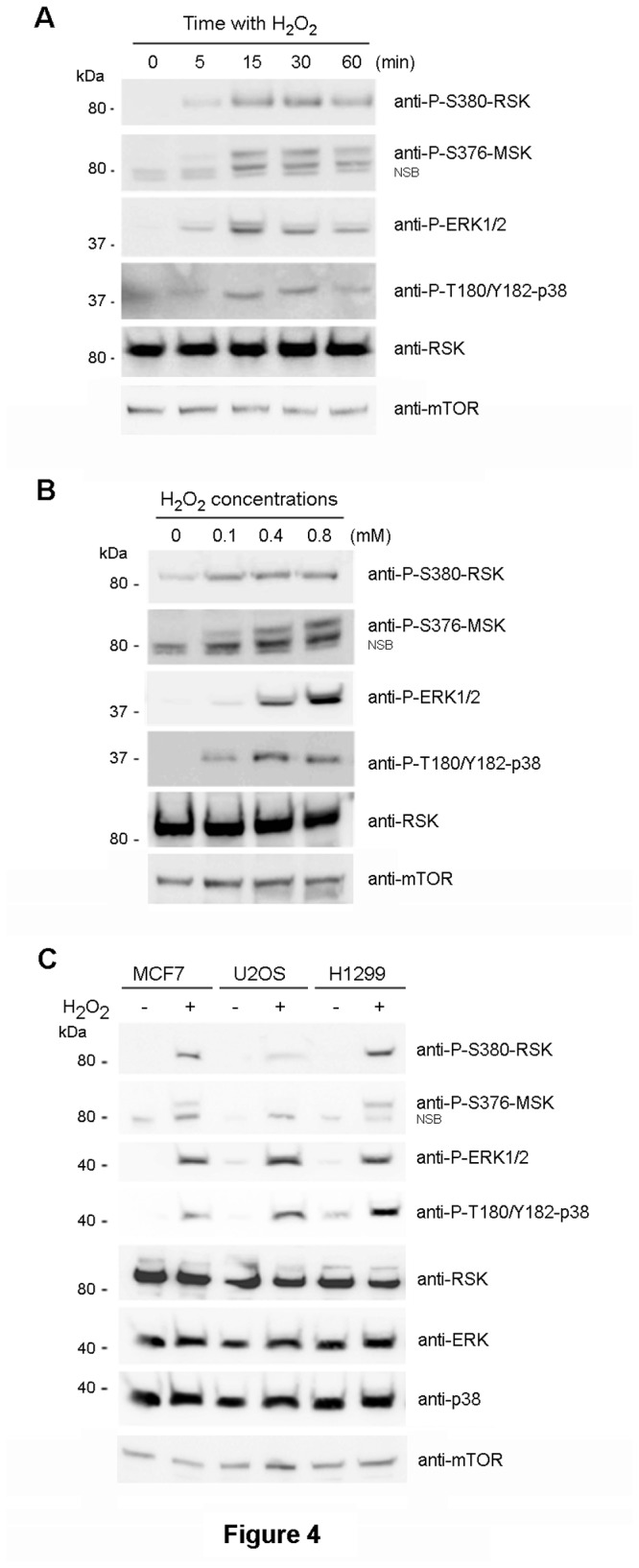
Activation of the MAPK signaling pathways in response to H_2_O_2._. Experiments of time and dose course were performed in MCF7 cells with 0.4 mM H_2_O_2_ (A) or for 30 min (B), respectively. Human cells were treated with 0.4 mM H_2_O_2_ for 30 min (C). Cell lysates were analyzed by Western blot with the indicated antibodies. NSB means non-specific band recognized by the antibody. Molecular weight markers are indicated on the left.

The previous data indicated that the phosphorylation of a p85 protein detected with anti-P-T389-S6K1 antibody in response to the oxidative stress mediated by H_2_O_2_ was independent of the mTOR/S6K1 signaling pathway. Thus, other pathways and proteins must be involved in this response. The MAPK signaling pathways are activated by incubation with H_2_O_2_. This activation is mediated by ERK and p38 kinases [7,20]. We had previously reported that these kinases also regulate amino acid signaling [17]. Under these conditions, ERK and p38 phosphorylate and activate RSK and MSK proteins in response to amino acids. RSK and MSK proteins are members of the family of serine /threonine kinases named AGC. S6K1 is also a member of this family. RSK and MSK proteins have a high degree of homology with the hydrophobic motif of S6K1, where T389 is located [16,17]. These structural similarities, together with the electrophoretic mobility of RSK and MSK proteins around 85-90 kDa and the previously shown cross-reaction of anti-P-T389-S6K1 antibody with phosphorylated RSK and MSK proteins, led us to check whether these proteins were phosphorylated in response to oxidative stress by H_2_O_2_. We had used antibodies against phosphorylated residues of RSK and MSK equivalents to T389 in S6K1, concretely anti-P-S380-RSK and anti-P-S376-MSK antibodies. We observed that RSK and MSK proteins were phosphorylated after incubation with H_2_O_2_ (Figure 4A/4B). The time and dose course was similar to that found with the anti-P-T389-S6K1 antibody (Figure 1). These effects were also observed in other human cells such as H1299 and, in less extension, in U2OS cells (Figure 4C). As positive control of the response to H_2_O_2_, we analyzed the activation of ERK and p38. As shown in Figure 4, phosphorylation of ERK and p38 correlated with the phosphorylation of RSK and MSK proteins.

**Figure 5:**
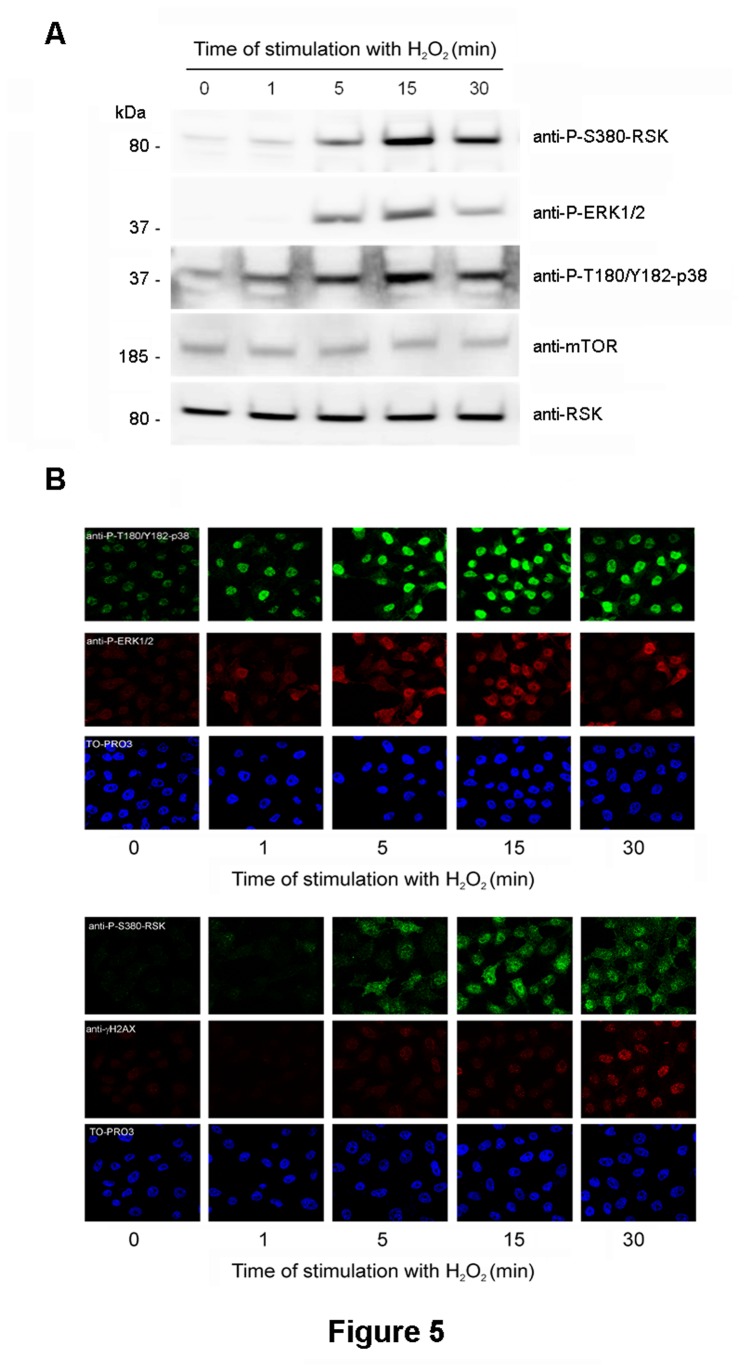
Nuclear translocation of phosphorylated ERK, p38 and RSK proteins. MCF7 cells were treated with 0.4 mM H_2_O_2_ for indicated times and analyzed by Western blot (A), as described in Figure 1, or by immunofluorescence (B) and using antibodies, as indicated in Experimental Procedures. Nuclear staining was detected with TO-PRO3.

Functional activation of the MAPK signaling pathways included the translocation to the nucleus of phosphorylated p38 and ERK. We analyzed these translocations in response to H_2_O_2_. As shown in Figure 5, a rapid nuclear translocation of phosphorylated p38 and ERK was observed at 1-5 min of incubation. Phosphorylation and translocation of RSK was also detected at 1-5 min of incubation. Phosphorylation of H2AX (γH2AX) in response to DNA damage was used as a positive control of treatment with H_2_O_2_. After 30 min, foci of γH2AX were clearly detected.

### Phosphorylation of p85 and S6 ribosomal proteins by H_2_O_2_ correlated with phosphorylation of RSK and MSK and were sensitive to inhibitors of the MAPK signaling pathways

**Figure 6:**
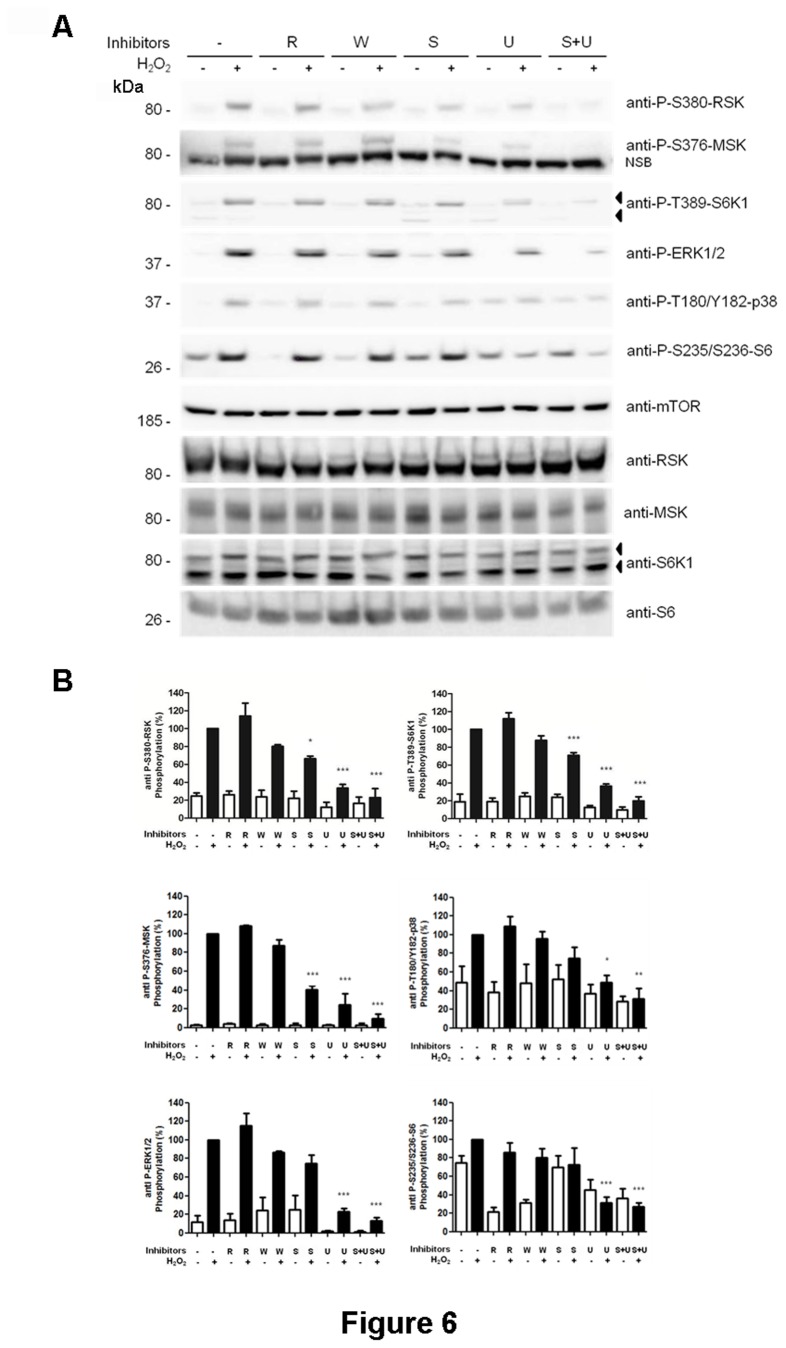
Phosphorylation of p85, RSK and MSK proteins was sensitive to inhibitors of the MAPK signaling pathways. (A) MCF7 cells were treated with 0.4 mM H_2_O_2_ for 30 min. Where indicated, cells were pre-incubated with 5 µM SB203580 (S), 5 µM U0126 (U), 100 nM wortmannin (W) or 20 nM rapamycin (R) for 60 min before treatment with H_2_O_2_. Cell lysates were analyzed by Western blot with the indicated antibodies. NSB means non-specific band recognized by the antibody. Molecular weight markers are indicated on the left. (B) Histograms represent the phosphorylation ratio of the indicated proteins. All bands were standardized with respect to mTOR levels. Values are the means ± SEM of the percentage of respective control for at least three independent experiments. Asterisks indicate values that are significantly different (*, p<0.05; **, p<0.01; ***, p<0.001) from the corresponding control value.

The previous data suggested that the phosphorylation of p85 protein detected with anti-P-T389-S6K1 antibody was the phosphorylation of RSK and MSK. Thus, it is would be expected that inhibition of the MAPK signaling pathway must inhibit phosphorylation of p85 protein in a similar manner to RSK and MSK. We checked this possibility. We performed experiments with H_2_O_2_ in the presence of known inhibitors of the MAPK signaling pathways (Figure 6). We observed that the phosphorylation of p85 protein was dependent on U0126, a specific inhibitor of ERK phosphorylation, and SB203580, a specific inhibitor of p38 activity. Interestingly, when both inhibitors were simultaneously used, phosphorylation of p85 protein was almost completely inhibited, suggesting crosstalk between ERK and p38 signaling (Figure 6). Similar results were obtained with the phosphorylation of RSK and MSK. As a positive control, phosphorylation of ERK and p38 was analyzed. Phosphorylation of S6 ribosomal protein was specifically inhibited by the ERK inhibitor, suggesting a specific role of this kinase or of a downstream kinase in this regulation. Under these conditions, the presence of rapamycin or wortmannin did not significantly affect the phosphorylation of ERK, p38, RSK or MSK proteins. None of these treatments modified endogenous levels of S6K1, RSK, MSK or S6 proteins (Figure 6). Levels of other proteins such as mTOR were not altered (Figure 6).

**Figure 7:**
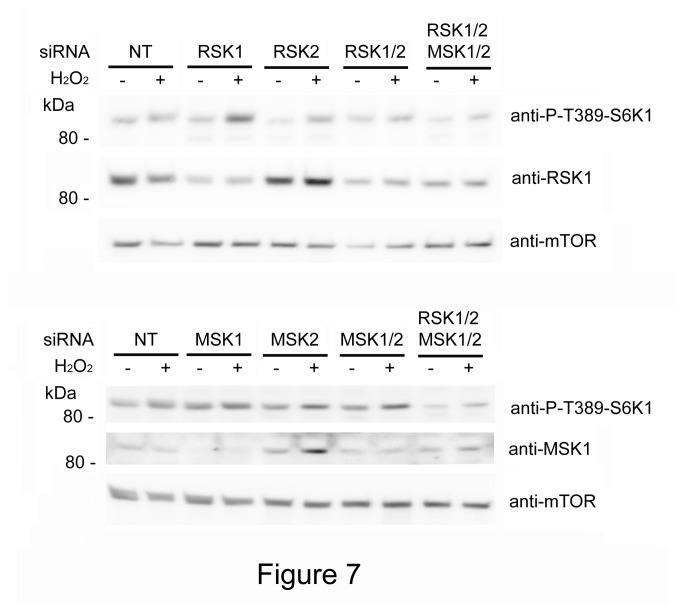
Anti-P-T389-S6K1 antibody detected RSK and MSK phosphorylated. MCF7 cells were transfected with the indicated siRNA 72 h before treatment with 0.4 mM H_2_O_2_ for 30 min. Cell lysates were analyzed by Western blot with the indicated antibodies. Molecular weight markers are indicated on the left. NT means non-targeting control.

To provide some evidence that p85 was likely RSK or MSK, knockdown experiments were performed. We used siRNA against the most abundant isoforms of RSK (RSK1 and RSK2) and MSK (MSK1/MSK2). The mix of siRNA against RSK or MSK isoforms decreased the p85 detection with anti-P-T389-S6K1 antibody (Figure 7). This decrease was more evident with simultaneous mix of siRNA against RSK and MSK isoforms. These results show that the anti-P-T389-S6K1 antibody was detecting RSK and MSK phosphorylated.

### Activation of RSK and MSK by H_2_O_2_

**Figure 8:**
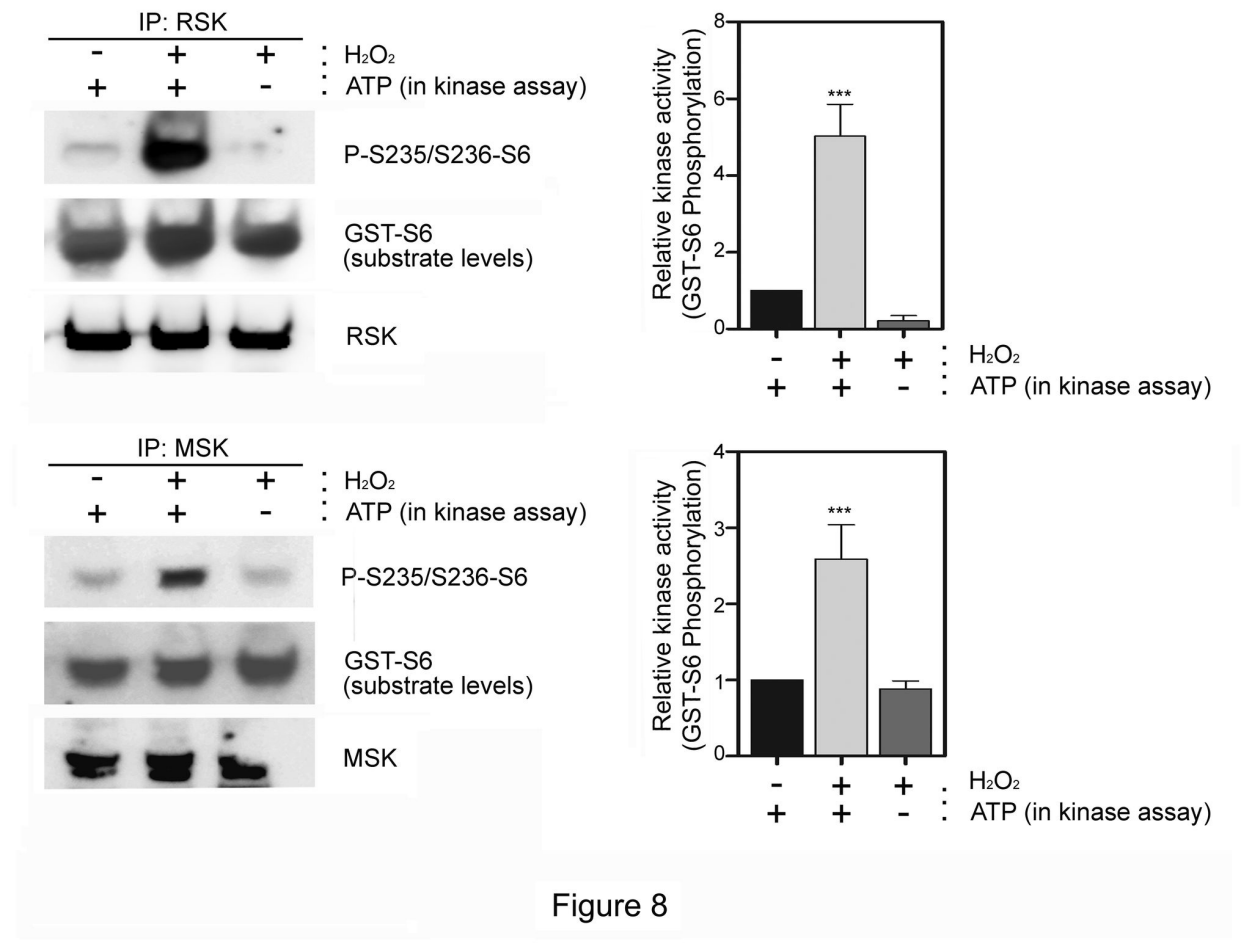
Activation of RSK and MSK by H_2_O_2_. *In* *vitro* kinase assay using RSK or MSK immunoprecipitates and purified GST-S6 as substrate. Lysates from MCF7 cells were immunoprecipitated with anti-RSK or anti-MSK antibodies (IP). Immunocomplexes were suspended in kinase buffer and incubated with purified GST-S6 in the presence or absence of ATP during 30 min at 30 °C. Reactions were stopped and the incorporation of phosphate was analyzed by immunoblotting using the anti-P-S235/S236-S6 antibody. Histograms: bands were normalized with respect to GST-S6 substrate levels detected with anti-GST antibody. Data represent the ratio of P-S235/S236-GST-S6 phosphorylation and are expressed as mean±SEM of percentage of respective control. Statistical analysis was carried out as indicated in Materials and methods.

The previous data suggest that the kinase activity of RSK and/or MSK is regulated by H_2_O_2_. To show this point, kinase assays were performed using purified kinases and GST-S6 fusion protein as substrate. As it is shown in Figure 8, the activities of RSK and MSK were stimulated by H_2_O_2_.

### p38α did not regulate phosphorylation of p70 S6K1 in response to the oxidative stress produced by H_2_O_2_

**Figure 9:**
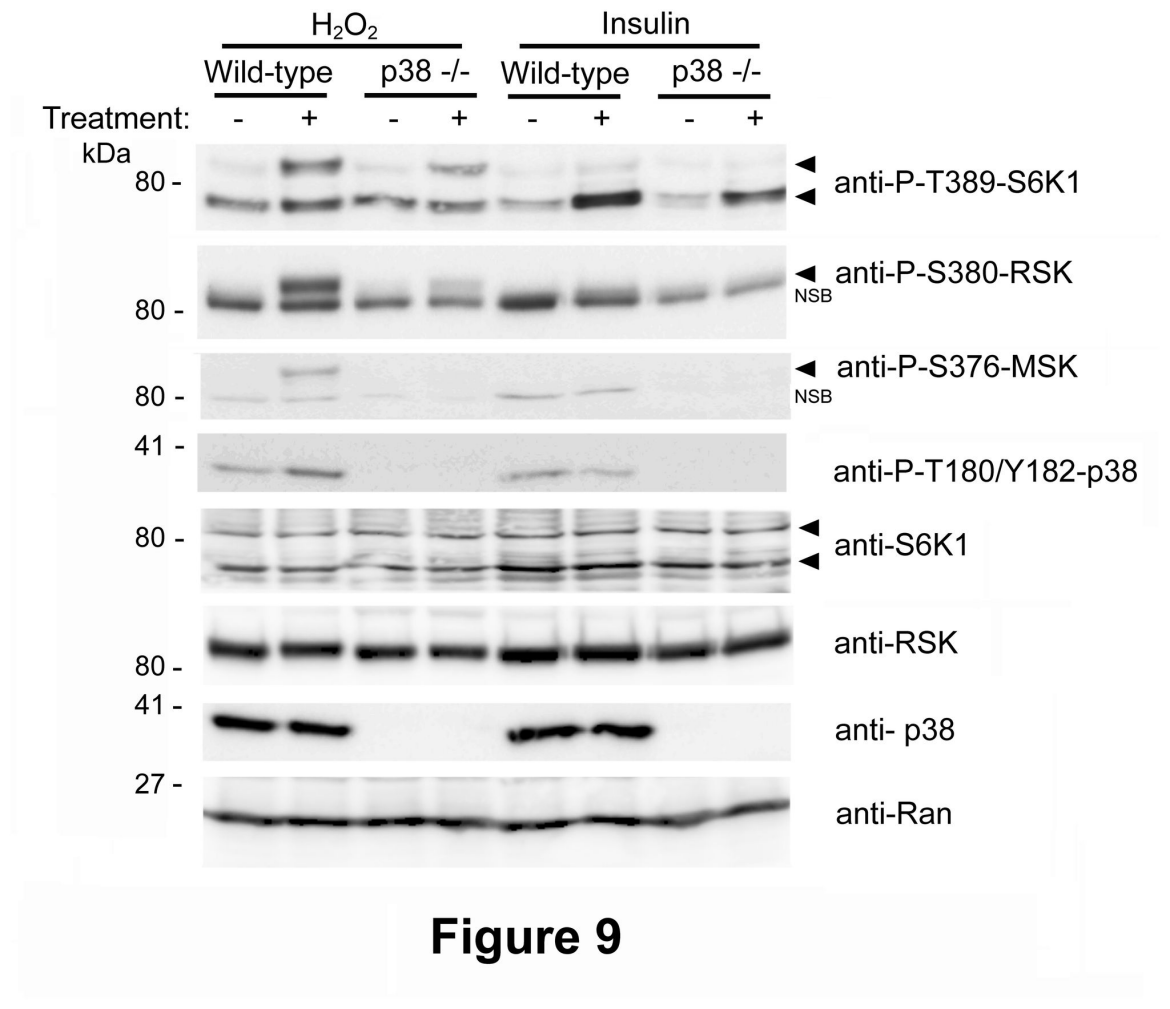
p38α did not regulate phosphorylation of p70 S6K1 in response to the oxidative stress caused by H_2_O_2_. WT and p38α-deficient MEFs were treated with 0.4 mM H_2_O_2_ for 30 min or deprived of serum overnight and then stimulated with 200 nM insulin for 30 min. Cell lysates were analyzed by Western blot with the indicated antibodies. NSB means non-specific band recognized by the antibody. Molecular weight markers are indicated on the left.

It has been reported that loss of p38α impairs mTOR/p70 S6K1 activation in response to H_2_O_2_ through Akt-independent mechanisms [12]. These experiments were performed in wild-type (WT) and p38α-deficient mouse embryonic fibroblasts (MEFs). Bearing in mind the above data, we analyzed S6K1 and MAPK signaling pathways in WT and p38α-deficient MEFs. To analyze the phosphorylation of mouse S6K1 p70 isoform, we used the well-shown activation and phosphorylation of p70 S6K1 by insulin as positive control. We observed an increase in the phosphorylation of p70 S6K1 after 30 min treatment with insulin (Figure 9). No significant changes were observed in the phosphorylation of RSK, MSK and p38 proteins due to insulin treatment. In parallel experiments, we compared the response of WT and p38α-deficient MEFs to treatment with H_2_O_2_ (Figure 9). Under these conditions and in line with the above data in human cells, increased phosphorylation of mouse p85 protein was observed in WT MEFs. Phosphorylation of mouse p70 S6K1 was not regulated in WT and p38α-deficient MEFs. Incubation with H_2_O_2_ activated the MAPK signaling pathway. Thus, phosphorylation of RSK, MSK and p38 proteins was seen in WT MEFs. In p38α-deficient cells, we observed a marked reduction in phosphorylation of RSK and MSK proteins (Figure 9). Phosphorylation of p85 protein was also reduced in p38α-deficient cells, suggesting that the anti-P-T389-S6K1 antibody detected phosphorylated RSK and MSK in mouse cells. Altogether, these results confirm our previous observations of human cells in response to the oxidative stress produced by H_2_O_2_.

## Discussion

The mTOR signaling pathway has an essential role in the regulation of mammalian growth and development. Hormones such as insulin and nutrients such as amino acids mediate their cellular effects through this pathway [21]. Several studies have analyzed the mTOR pathway in response to oxidative stress by H_2_O_2_. Most of these studies analyzed the activity of the mTOR complex 1 (mTORC1) through the analysis of S6K1 phosphorylation at T389 using anti-P-T389 antibodies. In some cases inhibition of mTORC1 activity was reported [11,14,15], whereas in others an increase was described [12,13,22]. These apparently contradictory results have been justified by the complexity of the mechanisms involved, cell type, H_2_O_2_ concentration and duration of the stress signal [12].

Stress conditions that produce DNA damage activate cell repair mechanisms where p53 activation is involved [1,23,24]. During p53 activation, inhibition of mTOR signaling has been observed [25]. Exposure of the cells to high H_2_O_2_ concentrations and/or during long time periods produces DNA damage and p53 activation. Thus, in these conditions, inhibition of the mTOR signaling pathway would be expected [11,14,15]. For low H_2_O_2_ concentrations or during shorter time periods, we showed that mTOR signaling was not involved and explained previous observations by the use of the anti-P-T389-S6K1 antibody from Cell Signaling. We showed that this antibody recognized a phosphorylated protein of 85 kDa in response to H_2_O_2_. Knockdown experiments together with the use of specific inhibitors let us show that this phosphorylated protein of 85 kDa was not regulated by mTOR and was not S6K1. A similar situation had been previously reported in signaling by amino acids, for which authors showed that the phosphorylated p85 protein recognized by the anti-P-T389-S6K1 antibody was phosphorylated RSK and MSK proteins [17]. To avoid misinterpretations in future experiments, we recommend that researchers using this anti-P-T389-S6K1 antibody check the correct size of the band detected and confirm their results with knockdown experiments. Simultaneous analysis with another well-known stimulus such as insulin also helps to detect the involvement of S6K1. We would also like to highlight that, since the S6 ribosomal protein may be a substrate of different kinases such as S6K1 or RSK [10,17], results using anti-P-S6 antibody to analyze mTOR/S6K1 signaling can be misinterpreted under conditions of activation of the MAPK signaling pathway.

Members of the MAPK family such as ERK, JNK or p38 are phosphorylated and activated in response to oxidative stress [1,7,20]. We confirmed the phosphorylation and activation (nuclear translocation) of ERK and p38 proteins by H_2_O_2_. Moreover, we reported the phosphorylation and activation of their substrates RSK and MSK. Interestingly, in human cells phosphorylation of both RSK and MSK proteins was sensitive to ERK and p38 activities, indicating that both kinases were necessary to phosphorylate RSK and MSK proteins completely in response to H_2_O_2_. In contrast, in mouse cells, phosphorylation of MSK by H_2_O_2_ seemed exclusively dependent on p38 since, in the absence of p38α, MSK phosphorylation was completely abolished. In this context, regulation was reported of mTORC1/p70 S6K1 by p38 in *Drosophila melanogaster* cells [13] and in MEF knockdown for p38α [12]. Using this last model, we showed that p70 S6K1 phosphorylation was stimulated by insulin, but not by H_2_O_2_. As expected, insulin did not activate the MAPK signaling pathways. Instead, oxidative stress activated phosphorylation of p38/ ERK/MSK in WT MEFs and of RSK in p38α-deficient MEFs. Anti-P-T389-S6K1 antibody detected the phosphorylated p85 protein and its regulation correlated with phosphorylated RSK and MSK proteins. All these observations suggest that, at least in mouse and in human cells, the fast response to oxidative stress caused by low concentrations of H_2_O_2_ is mediated by MAPK signaling pathways and not by the mTORC1/p70 S6K1 signaling pathway. In summary, we believe that this report helps to explain previous controversial results and to clarify the cellular signaling activated in response to oxidative stress.
