# Correction: Contribution of S6K1/MAPK Signaling Pathways in the Response to Oxidative Stress: Activation of RSK and MSK by Hydrogen Peroxide

**DOI:** 10.1371/annotation/0b485bd9-b1b2-4c60-ab22-3ac5d271dc59

**Published:** 2013-11-06

**Authors:** Anna Siebel, Monica Cubillos-Rojas, Roberto Christ Santos, Taiane Schneider, Carla Denise Bonan, Ramon Bartrons, Francesc Ventura, Jarbas Rodrigues de Oliveira, Jose Luis Rosa

There is an error in the byline noting the equal contribution of the authors. Only authors Anna Siebel and Monica Cubillos-Rojas are equal contributors. 

